# Coping Strategies, Ratio of Positive to Negative Affect, and Burden in Adult Children of Parents With Memory Loss

**DOI:** 10.1093/geroni/igaf122.639

**Published:** 2025-12-31

**Authors:** Dustin Gad, Jenna Wells, Joan Monin, Kristie Wood

**Affiliations:** Cornell University, Ithaca, New York, United States; Cornell University, Ithaca, New York, United States; Yale, Yale, Connecticut, United States; University of Colorado Anschutz Medical Campus, Aurora, Colorado, United States

## Abstract

Caring for an individual with memory loss is associated with high levels of burden, leading to adverse health effects. Caregivers use behavioral and cognitive coping strategies to manage this stress. Approach coping strategies (e.g., seeking emotional support) are generally better for caregivers’ mental health than avoidant coping strategies (e.g., denial). Engaging in less adaptive coping strategies may fuel decreases in caregivers’ positive emotions and increases in their negative emotions, thereby amplifying burden. We evaluated whether caregivers’ coping strategies (Brief COPE) are associated with their levels of burden (cross-sectionally and longitudinally over one year; ZBI-12); and whether these associations are moderated by ratios of positive to negative affect (calculated by dividing their reported positive emotions by their negative emotions; PANAS) in adult-child caregivers (N = 150) of parents with memory loss. In a multiple regression, greater use of approach coping (b= .14, p= .031) and avoidant coping (b= .59, p< .001) were associated with higher burden. Further, greater avoidant coping at baseline was associated with greater increases in burden one year later (b= .31, p= .001), accounting for baseline burden. No significant association was found between approach coping and changes in burden. Ratios of positive to negative affect moderated the cross-sectional association between avoidant coping and burden such that caregivers who reported lower ratios of positive to negative affect reported higher levels of burden (b= .51, p= .007). Although avoidant coping strategies are often associated with poor outcomes, caregivers who experience more positive emotions relative to their negative emotions may be buffered.

